# Enhancing validity, reliability and participation in self-reported health outcome measurement for children and young people: a systematic review of recall period, response scale format, and administration modality

**DOI:** 10.1007/s11136-021-02814-4

**Published:** 2021-03-18

**Authors:** L. Coombes, K. Bristowe, C. Ellis-Smith, J. Aworinde, L. K. Fraser, J. Downing, M. Bluebond-Langner, L. Chambers, F. E. M. Murtagh, R. Harding

**Affiliations:** 1 King’s College London, Florence Nightingale Faculty of Nursing, Midwifery and Palliative Care, Cicely Saunders Institute, London, UK; 2 Royal Marsden NHS Foundation Trust, London, UK; 3 Martin House Research Centre, Department of Health Sciences, University of York, York, UK; 4 International Children’s Palliative Care Network, Kampala, Uganda; 5 UCL Great Ormond Street Institute of Child Health, Louis Dundas Centre for Children’s Palliative Care, University College London, London, UK; 6 Rutgers University, Camden, NJ USA; 7 Together for Short Lives, Bristol, UK; 8 Wolfson Palliative Care Research Centre, Hull York Medical School, University of Hull, Hull, UK

**Keywords:** Child, Outcome Assessment, Healthcare, Psychometrics, Cognition, Questionnaire

## Abstract

**Introduction:**

Self-report is the gold standard for measuring children’s health-related outcomes. Design of such measures is complex and challenging. This review aims to systematically appraise the evidence on recall period, response scale format, mode of administration and approaches needed to enable children and young people < 19 years to participate in valid and reliable self-reporting of their health outcomes.

**Method:**

PsycInfo, Medline, CINAHL and Embase were searched from 1 January 1990 to 15 March 2020, and citation searching undertaken in Scopus. Articles were included if they were primary research or case reports of ≥ 3 participants reporting the following: recall period, response scale selection, administration modality. Quality was assessed using QualSyst, and results synthesised narratively. This review was conducted and reported according to PRISMA guidelines.

**Results:**

81 of 13,215 retrieved articles met the inclusion criteria. Children < 5 years old cannot validly and reliably self-report health outcomes. Face scales demonstrate better psychometric properties than visual analogue or Likert scales. Computerised and paper scales generally show equivalent construct validity. Children prefer computerised measures. Children ≤ 7 years old think dichotomously so need two response options. Those > 8 years old can reliably use a 3-point scale.

**Conclusion:**

The results of this review have both clinical and research implications. They can be used to inform appropriate choice of PROM for use with CYP in the clinical setting. We also give eight recommendations for future development of self-reported outcome measures for children and young people.

**Supplementary Information:**

The online version contains supplementary material available at 10.1007/s11136-021-02814-4.

## Introduction

Patient-reported outcome measures (PROMs) are validated questionnaires that are completed by patients to ascertain perceptions of their health status and well-being [1, 2]. PROMs range from single-item symptom ratings e.g., pain scales, to complex multidimensional tools measuring health-related quality of life [3]. PROMs are considered to be the gold standard for measuring subjective experiences, because the information comes directly from the patient [4]. When collecting data on the health-related outcomes of children and young people (CYP) it is good practice to enable CYP to self-report whenever possible.

The design and implementation of PROMs for CYP presents methodological complexities, including consideration of response format, recall period and the mode of administration [5, 6]. These considerations should be addressed at the design stage to ensure PROMS are both feasible (*ability* to complete a measure) and acceptable (*willingness* to complete a measure) [7]. Acceptable modes of administration are crucial to enable CYP to engage and provide valid and reliable results [8].

Careful consideration of recall period, response scale format and administration modality during all stages of PROM design may increase response and completion rates, whilst maintaining and enhancing validity and reliability. The aim of this review is to systematically appraise the evidence on response scale type, recall period, administration modality and approaches to enable CYP < 19 years to participate in valid and reliable self-reporting of their health outcomes.

## Methods

This systematic literature review was conducted and reported in accordance with the Preferred Reporting Items for Systematic Reviews and Meta-Analyses (PRISMA) guidelines [9], and registered on PROSPERO (CRD42019135264).

PsycINFO, Medline, CINAHL and Embase were searched from 1st January 1980 (i.e., when outcome measurement in children began to be reported in the scientific literature [10–12]) to 15th March 2020. The search combined terms for children used in a previous systematic review [13] with those for different response scale formats, recall periods and methods of administration ( \* MERGEFORMAT Table 1 Search terms). Additional articles were searched using ‘cited by’ (Scopus), forwards and backwards referencing and consulting other experts in the field. The full Medline search strategy is reported in Supplementary Appendix 1.

**Table 1:** Search terms

Children	Recall Period	Response format	Administration mode
Exp child/or exp p?ediatrics/or child* or (adolescen* or p?ediatric* or youth* or juvenile or teen* or young people or schoolchild* or school age* or kid*)	Recall period or recall interval or patient recall or recall bias	Response scale or likert scale or visual analog* scale or VAS or numerical rating scale or verbal rating scale or faces scale or dichotomous scale or yes no response or response option*	(Outcome measure adj2 (paper or (paper and pen) or tablet or tablet computer or app or application or telephone or face to face or internet) or (measure adj2 (paper or (paper and pen) or tablet or tablet computer or app or application or telephone or face to face or internet) or (scale adj2 (paper or (paper and pen) or tablet or tablet computer or app or application or telephone or face to face or internet) or (questionnaire adj2 (paper or (paper and pen) or tablet or tablet computer or app or application or telephone or face to face or internet) or (survey adj2 (paper or (paper and pen) or tablet or tablet computer or app or application or telephone or face to face or internet)
Combined with ‘and’			

### Inclusion and exclusion criteria

Inclusion criteria were: (1) study population CYP ≤ 18 years old (studies reporting participants ≥ 19 years old were included if data were presented separately). Our original protocol planned to include those ≤ 17 years old but a large proportion of identified papers included 18 year olds so this was amended; (2) primary research of self-report of health outcomes among CYP; (3) studies evaluating recall period, response format, administration modality or approaches to engage CYP in self-reporting health outcomes in terms of their effect on measurement properties (validity, reliability and responsiveness) [7], acceptability (willingness to use a particular response format, administration mode or recall period), feasibility of use (ability to use a particular response format, administration mode or recall period) or preference for a particular mode, response format or recall period [7]; (4) written in the English language.

Exclusion criteria were case reports of < 3 participants (due to the risk of selection bias), discussion articles, editorials, reports, letters and reviews.

### Study selection and data extraction

Citations were imported to EndNote (v9) and de-duplicated. Titles and abstracts of retrieved articles were screened for eligibility by one reviewer (LC). If there was not enough information within the title and abstract to determine eligibility, the full text article was screened. Remaining full text articles were screened by LC. 10% of the full text articles were screened by a 2nd reviewer (JA). Any discrepancies were resolved through discussion, and a third reviewer consulted as necessary (CES or RH).

Data from eligible studies were extracted into a common table: study authors, year of publication, geographic location, objective, study design, sample characteristics (population, size, setting), measure characteristics reported (recall period, response format, administration modality) and main findings.

### Quality appraisal and data synthesis

QualSyst was applied rather than the COSMIN checklist in line with the overall aim of this review to examine response format, administration mode and recall period, rather than to appraise specific PROMs [14]. QualSyst assesses study quality with two scoring systems, one for qualitative and one for quantitative research. The qualitative scale consists of ten items with scores from zero to two, yielding a maximum score of 20. The quantitative scale consists of 14 items with scores from zero to two, an option to score an item ‘not applicable’, and maximum total score of 28. Overall scores are reported as percentages. Mixed method studies received two scores—one each for the qualitative and quantitative components [15]. Inter-rater agreement was assessed for 10% of the included articles.

Results were synthesized narratively to appraise the heterogeneity of included studies, and any similarities or differences in findings. The results were used to make recommendations on recall period, response format and administration mode when developing self-reported health outcome measures for CYP.

## Results

### Study selection

**Fig. 1:**
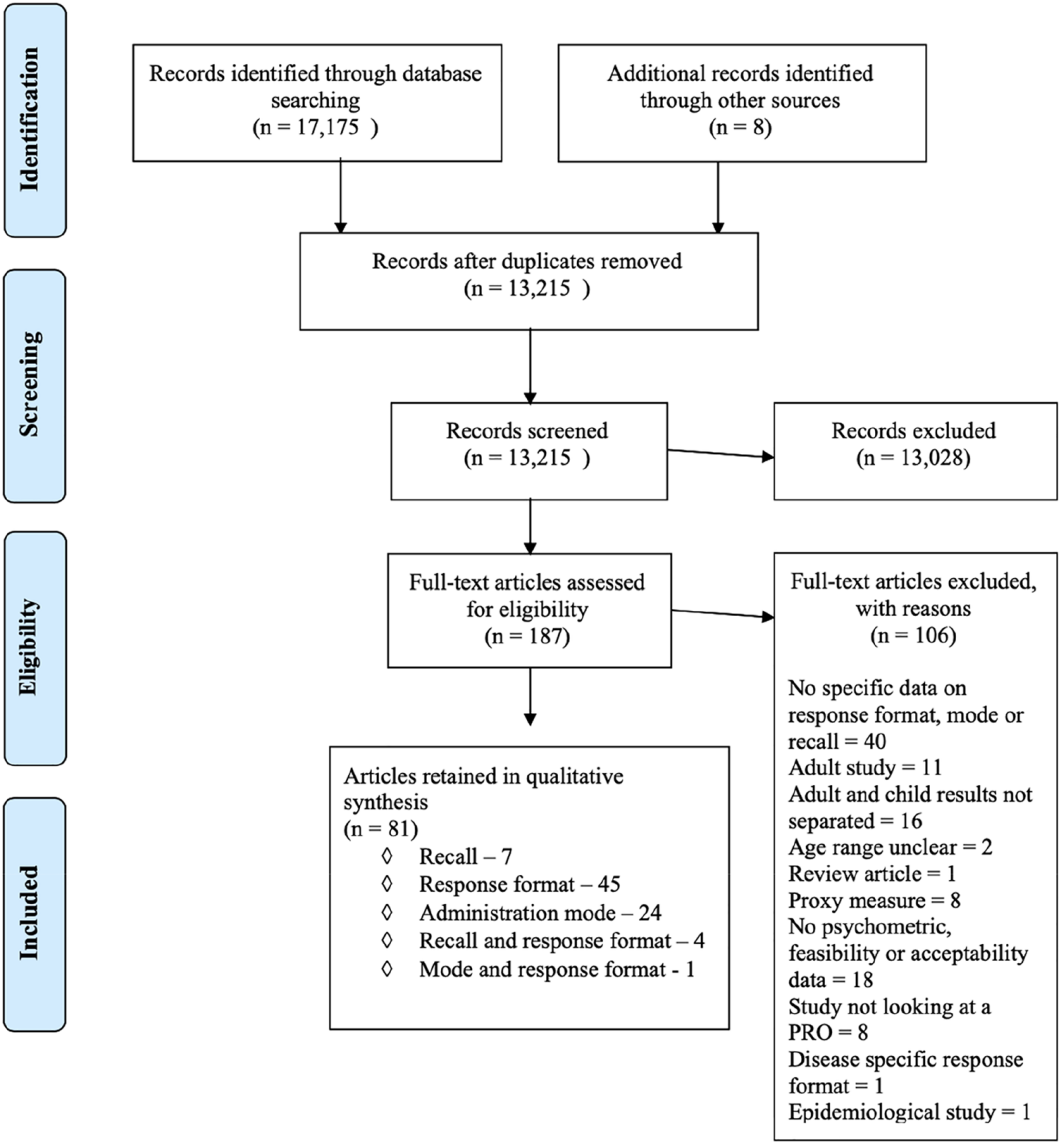
PRISMA flowchart of study selection process [9]

The search identified 13,207 articles after deduplication. A further 8 were identified via reference searching. 187 articles required full text review and 81 met the inclusion criteria. Of the articles included, 45 reported on response format [16–60], seven on recall period [61–67], 24 on administration mode [68–91], four on both recall and response format [92–95] and one on response format and administration mode [96]. The PRISMA flowchart is shown in Fig. 1 [9].

### General Information on Included Studies

Tables 2, 3 and 4 summarise included studies and quality scores. Supplements 2 and 3 provide details of quality scores by item. The majority of included studies were conducted in Europe (*n* = 25/81) [17–20, 22, 26, 34, 37, 40, 41, 44, 50, 59, 65, 69, 71, 72, 75–78, 82–84, 87], the USA (*n* = 31/81) [16, 28, 29, 36, 38, 46–48, 51, 53, 54, 57, 58, 61–64, 66–68, 70, 74, 79, 80, 85, 86, 89, 90, 92, 95, 96] and Canada (*n* = 18/81) [21, 23–25, 27, 32, 39, 42, 43, 49, 52, 55, 56, 60, 81, 88, 91, 93] with two from Australia [31, 33], and one each from Japan [45], Korea [35], New Zealand [73], Kenya [94] and Jordan [30]. With respect to study design, *n* = 68/81 used quantitative methodology, *n* = 11/81 qualitative methodology and *n* = 2/81 mixed methods. Settings were predominantly home, school/nursery or hospital, and the 33,834 participants ranged from 3 to 18 years and were either healthy children (*n* = 30) or had one of a wide range of medical conditions (*n* = 50).

**Table 2:** Summary of studies on response format

Author (date); Country; Study Design; Measurement properties evaluated	Objective	Sample size (*N*); Setting; Age; Population	Main findings	QualSyst Score (%)
Baxter (2011) [16]; USA; Quantitative; Prospective; Feasibility, construct validity, responsiveness	To create and validate a pictorial scale with regular incremental levels between scores depicting increasing nausea intensity (BARF scale)	*N* = 127; Hospital; 7–18 years; Emergency department and surgery	The Spearman correlation coefficient of the first paired BARF and VAS for nausea scores was 0.93. VAS for nausea and BARF scores (*P* = .20) were significantly higher in patients requiring antiemetic agents and decreased significantly after treatment, while posttreatment pain scores (*P* = .47) for patients receiving only antiemetic agents did not. All patients understood the pictorial faces scales	68
Benson (2016) [17]; UK; Quantitative; Prospective; Construct validity	To test items, identified through previous qualitative interviews, that might form the basis of a new Malocclusion Impact Questionnaire for young people	*N* = 184; Hospital; 10–16 years ; Dental outpatients	Using Rasch analysis it was shown that all but one item had disordered thresholds, indicating response categories were not functioning as expected. The original 5-point response scale format was reduced to 3 points	60
Berntson (2001) [18]; Sweden; Quantitative; Cross-sectional; Acceptability, construct validity reliability	To evaluate the concordance between pain assessments made on a VAS^a^ and a 4-point verbal descriptor scale and establish scale preference	*N* = 12; Hospital; 10–18 years; juvenile arthritis	Slight pain on verbal scale corresponded to a wide interval of 7–65 on VAS suggesting VAS was difficult to interpret. Preference was for VAS (69%) but this did not show the most reliable results	68
Borgers (2003) [19]; Netherlands; Quantitative; Prospective; Feasibility, reliability	To investigate the effects of partially labelled response options and vague quantifiers in response stability compared to completely labelled response options and the use of clearly quantified words in children of different ages	*N* = 91; Home; 8–16 years; Healthy	No effect on stability over time was found when offering vague quantifiers in the response options (p > 0.05). Young children do not benefit from the extra information of completely labelled response options. Offering different types of response option can lead to substantially different structural models	75
Borgers (2004) [20]; Netherlands; Quantitative; Prospective Feasibility, reliability	To examine the effects of negatively formulated questions, number of response options and offering a neutral midpoint as response option question characteristics on the reliability of responses	*N* = 222; Home; 8–16 years; Healthy;	Negatively formulated questions had no effect on reliability, although children respond consistently differently to negatively formulated questions as opposed to positively formulated ones. Offering about 4 response options is optimal (reliability increased up to 6, more than 7 caused a decrease)	80
Campbell (2011) [21]; Canada; Quantitative; Cross-sectional; Feasibility	To investigate the utility of a VAS^a^ to measure peer conflict resolution knowledge in children with language impairment (LI) and typically developing peers (TLD). Are children with varying language status able to express nuances in social knowledge by marking responses along the full VAS	*N* = 26; School; 9–12 years; Healthy	Those with TLD used the whole VAS; most (83%) with LI relied solely on scale anchors	59
Castarlenas (2013) [22]; Spain; Quantitative; Cross-sectional; Acceptability, construct validity	To assess whether the NRS-11^b^ is a valid tool with 6–8 year old children when presented verbally	*N* = 126; School; 6–8 years; Healthy	The NRS-11 showed high convergent construct validity with VAS^a^, FPS-R^c^ and CAS^d^ (r = 0.73–0.86), adequate discriminant validity (z = 2.05–5.55) and adequate criterion validity (r = 0.45–0.70). Preference order = CAS > NRS > FPS-R > VAS	75
Chambers (1998) [23]; Canada; Quantitative; Cross-sectional; Feasibility	To examine the potentially biasing impact of neutral or smiling face as a no pain anchor on children’s reports of pain in response to a series of vignettes	*N* = 100; Childcare centres; 5–12 years; Healthy	Children who use a smile anchored scale had significantly higher pain scores for no pain and pain negative emotions (p < 0.001) and significantly lower faces pain scores for pain/positive vignettes than children who use a neutral anchored face scale (p < 0.001). Faces scales that use smiling anchors may confound affective states with pain ratings	63
Chambers (1999) [24]; Canada; Quantitative; Cross-sectional; Acceptability, feasibility	To examine the potential for bias in children’s’ self-report of pain when using scales with smiling rather than neutral anchors and to establish preference of type of faces scale	*N* = 75; Hospital; 5–12 years ; Endocrine/diabetes	Scores across scales were highly correlated (r = 0.81–0.93). There was no age or gender interaction effect. Pain was rated significantly higher when scales with a smiling, rather than neutral, anchor were used (p = 0.001). 52.1% of children preferred scales they perceived to be happy and cartoon-like	75
Chambers (2005) [25]; Canada; Quantitative; Cross-sectional; Acceptability, feasibility	To determine whether scales beginning with a smiling rather than neutral “no pain” face would produce higher ratings in the assessment of postoperative pain intensity in children and to compare ratings using different faces. Preference also asked	*N* = 78; Hospital; 5–13 years ; Post-surgical	Children’s ratings of postoperative pain intensity are influenced by the presence of smiling “no pain” face at the beginning of faces scales, with such scales producing significantly higher ratings than scales with neutral “no pain” faces (p < 0.01). Ratings on the independent CAS^d^ measure were more comparable to those provided on faces scales with neutral “no pain” faces. 55.6% preferred Wong Baker faces scale despite it giving the highest pain scores	83
Decruynaere (2009) [26]; Belgium; Quantitative; Cross-sectional; Construct validity, feasibility	To examine with the rating scale model how a sample of healthy children from 4–7 distinguish different faces when rating imaginary painful situations	*N* = 121/76; Schools and sports centres; 4–7 years ; General	Children performed better on a 3-point faces scale than 6-point scale. Ability improves with age on a 3-point faces scale. 4–5-year-olds could only distinguish 2 response categories	70
Emmott (2017) [27]; Canada; Quantitative; Cross-sectional; Construct validity, feasibility	To evaluate validity and feasibility of 2 simplified pain scales—S-FPS and S-COS in pre-school age children	*N* = 180; Hospital; 3–6 years ; Venepuncture	The ability to discriminate pain vs no pain was improved with S-FPS^d^ and S-COS^f^ (p = 0.858) compared with FPS-R^c^ (p = 0.036 with S-FPS and p = 0.022 with C-COS) within 4–6-year olds but not 3-year olds. Quantitative pain rating remains challenging for 3-year-olds	88
Fanciullo (2007) [28]; USA; Quantitative; Cross-sectional; Acceptability, construct validity, feasibility	To determine initial psychometric properties and feasibility of a new Computer Face Scale for measuring children’s pain	*N* = 54; Hospital; 3–17 years ; Hospitalised in pain/healthy	76% of children from3 years preferred moveable online faces to select their degree of pain over paper and pen static faces. Paired t tests showed significantly more hospitalised children reported pain than non-hospitalised (p < 0.001). Correlation with Wong-Baker faces scale r = -0.72	75
Fritz (1994) [29]; USA; Quantitative; Prospective; Feasibility	To determine whether the use of pictorial anchors improved a VAS^a^ designed to assess asthma episodes	*N* = 77; Summer camp; 8–15 years; Asthma	The mean VAS scores increased by 64% using the pictorial VAS while the mean PEFRs^g^ in the 2 years were almost identical, suggesting that changes on the VAS were not due to differences in pulmonary functioning. For boys, the increase in individual mean VAS score in year 2 using the pictorial VAS was 44%; for girls, the increase in individual mean VAS scores was 112%. Use of a pictorial anchor led to greater usage of the full range of the scale	77
Gharaibeh (2002) [30]; Jordan; Quantitative; Cross-sectional; Acceptability, construct validity, reliability	To test the reliability and cultural validity of the following three pain assessment scales: Faces Scale, the Word Description Scale, and the Poker Chip Scale	*N* = 95; Hospital; 3–14 years; Venepuncture	55.8% of children preferred the Poker Chip Tool to the Faces Scale and the Word Descriptive Scale. There was significant convergent validity (p < 0.01) and test–retest reliability (p < 0.01) between the three scales	60
Goodenough (1997) [31]; Australia; Quantitative; Cross sectional; Construct validity, feasibility	To compare the utility of the Faces Pain Scale with three other self-report measures (VAS^a^, Poker Chip, VRS^h^) of pain severity. These four scales were compared and contrasted in terms of the facility of application and comprehension by young children and their relative response frequency distributions	*N* = 50; Hospital; 4–7 years; Immunisation	Scores on all 4 scales correlated well (r > 0.7). The scales seemed to be measuring the same construct of pain. The faces scale was well understood. 12% had difficulty with the Visual analogue toy scale. The faces pain scale was skewed to low possibly because there are too many response options for the age group causing them to choose the extreme options	60
Gulur (2009) [58]; USA; Quantitative; Prospective; Acceptability, construct validity, reliability	1) to determine whether children understood the link between the facial expressions of smiling and frowning and the subjective feelings of happiness and pain/hurt. 2) to determine whether children understood that relative degrees of smiling or frowning were linked to relative degrees of happiness and pain/ hurt. 3) to determine the concurrent validity of the Computer Face Scale with the Wong-Baker Faces Scale. 4) to determine the test–retest reliability of the Computer Face Scale	*N* = 79/50; Hospital; 3–17 years; Study 1 Surgical; Study 2 general inpatients	The computerised scale showed concurrent validity with Wong-Baker faces (r = -0.68). 15-min test–retest reliability r = 0.77. 77% preferred the computerised faces scale. Participants were able to use both scales	45
Gupta (2016) [96]; USA; Qualitative; Cross-sectional; Acceptability, feasibility	To evaluate comprehension and usability of a modified electronic version of the electronic version of the FPS-R^c^ for children aged 4–17 years with sickle cell disease	*N* = 22; Unclear; 4–17 years; Sickle cell	Children age 4–6 years were generally unable to demonstrate understanding of the FPS-R and its response scale. Children > 7 years understood the scale and could complete it electronically. Those aged 7–8 years often needed parental assistance	55
Hicks (2001) [32]; Canada; Quantitative; Cross-sectional; Construct validity	1) to revise the FPS^i^ from 7 faces to 6 to make scores comparable to other measures (0–5 or 0–10). 2) to evaluate the validity of the revised version	*N* = 76/45; Ear piercing/Hospital; 4–12 years; Healthy/hospital	The validity of the revised scale is supported by a strong positive correlation (r = 0.93) with a VAS^a^ measure in healthy children aged 5–12 years. In hospitalised children the revised scale correlated with the VAS (r = 0.92) and CAS^d^ (r = 0.84)	60
Hunter (2000) [33]; Australia; Quantitative; Cross-sectional, Feasibility	To further investigate the psychometric properties of the faces pain scale	*N* = 135; School; 3.5–6.5 years; Healthy	All children were capable of making meaningful discriminations. Children had difficulties with the middle of the scale suggesting that it formed an acceptable series but could not be considered an interval scale. The scale is best reserved for school age children	50
Irwin (2009) [95]; USA; Qualitative; Cross-sectional; Feasibility	To conduct cognitive interviews with children and adolescents to gain feedback on items measuring physical functioning, emotional health, social health, fatigue, pain and asthma specific symptoms for PROMIS item bank	*N* = 77; Hospital/community; 8–17 years; Healthy/asthma	Response options were understood by the majority of participants (up to 5 options). Children could clearly identify variable levels of functioning. Younger children misunderstood the word difficulty, so it was changed to trouble	65
Joffer (2016) [34]; Sweden; Qualitative; Cross-sectional; Feasibility	To explore how adolescents interpret and reason when answering a question on self-rated health	*N* = 58; School; 12–18 years; Healthy	Participants’ understandings of the response alternative “Neither good, nor bad” varied. Some regarded it as normal and “in the middle”, some as a negative alternative, and others as a passive state. The five response options all demonstrated differences in self-rated health	60
Jung (2018) [35]; Korea; Quantitative; Prospective; Construct validity	To develop and validate the “Pain Block” concrete ordinal scale for 4- to 7-year-old children. Psychometric properties were compared with the FPS-R^c^	*N* = 163; Hospital; 4–7 years; Emergency dept	Agreement between the 2 pain scales was acceptable, with 95% of the values within the predetermined limit (r = 0.82). The pain scores for both pain scales were significantly decreased when analgesics or pain-relieving procedures were administered (p < 0.001). The Pain Block pain scale could be used to assess pain in 4- to 7-year-old children capable of understanding and counting up to the number 5, even if they do not understand the FPS-R pain scale	68
Keck (1996) [36]; USA; Quantitative; Prospective; Acceptability, construct validity, reliability	To investigate the Faces and modified Word Descriptor Scale for concurrent validity, discriminant validity and test retest reliability	*N* = 118; Hospital; 3–18 years; Haematology and oncology; venepuncture	Both the word descriptor and faces scales demonstrated discriminant validity (p < 0.001 for scores before and after painful procedure) and concurrent validity (r > 0.71) and test–retest reliability (faces r = 0.9 and word scale r = 0.92). All children understood the scales. The majority of children in all age groups preferred the faces scale (65.1%)	50
Klassen (2015) [60]; Canada; Mixed methods; Cross-sectional; Acceptability, construct validity, feasibility, reliability,	1) to conduct individual cognitive interviews with adolescents age 12–18 with different health conditions to obtain their feedback on the instructions, response options and items of a transition questionnaire (Transition-Q) with a 5-point Likert response option and to identify any missing content and to revise the scale as necessary. 2) conduct a large-scale field test to examine reliability and validity	*N* = 32/37; Hospital; 12–18 years; Chronic conditions	Item response option thresholds weren’t ordered for 13 of 18 items. Items were rescored in to 3 response options. 14 participants did not like the agree/disagree response format. It was changed to frequency (never, sometimes, often and always). This was preferred by 8/9 in the second round. Cronbach’s ⍺ = 0.85. Test–retest reliability = 0.9	90 (quant) 55 (qual)
Lawford (2001) [37]; UK; Quantitative; Cross-sectional; Feasibility, reliability	To provide an empirical basis for selecting the response format of a QOL measure for 3–8-year olds (4 point Likert scale vs 4 coloured circles)	*N* = 28; Nursery school; 4–5 years; Healthy	The Likert scale took significantly longer to complete (p < 0.005). The coloured circle format had higher internal consistency than the Likert scale (⍺ = 0.7 vs 0.48)	65
Leske (2015) [38]; USA; Quantitative; Cross-sectional; Construct validity	To use Rasch analysis to refine the Intermittent Exotropia Questionnaire, removing items that do not contribute meaningful information and ensure response options are properly interpreted	*N* = 575; Eye clinics; 8–17 years; Intermittent exotropia	Performance of the child and adult versions were enhanced by reducing the number of response options from 5 to 3	80
Locker (2007) [39]; Canada; Quantitative; Cross-sectional; Construct validity	To assess the performance of negatively and positively worded items in questionnaires to measure child perceptions of child oral health-related quality of life	*N* = 91; Dental clinics; 10–14 years; Dental/oro-facial	Positively worded items elicited significantly more ‘don’t know’ responses and missing values. The performance of positively worded items was unsatisfactory	85
Luffy (2003) [57]; USA; Quantitative; Cross-sectional; Acceptability, construct validity, reliability	To compare the validity, reliability and preference of pain intensity measurement tools—the African American Oucher scale, Wong-Baker Faces scale and VAS^a^	*N* = 100; Outpatient clinics; 3–18 years; Sickle cell	Faces and African American Oucher are valid (no significant difference in scores between Oucher and Wong-Baker faces) and reliable (test–retest p < 0.005) tools for measuring pain in children. The VAS was not. 56% preferred the faces scale	50
Maïano (2009) [40]; France; Quantitative; Cross-sectional; Construct validity, reliability	To test the factor validity and reliability of 2 versions (graphical scale vs Likert scale) of the Very Short Form of the Physical Self-Inventory (PSI-VSF), with a sample of adolescents with mild to moderate intellectual disability	*N* = 342; School; 12–18 years; Learning difficulties	Both versions showed good structural validity, with the graphical version being superior. The graphical faces scale version had higher internal consistency (⍺ = 0.7–0.74 vs 0.65–0.67) than the Likert scale	80
McGrath (1996) [55]; Canada; Quantitative; Cross-sectional; Construct validity, feasibility	To determine the validity of the CAS^d^ as a pain measure for children by evaluating the psychometric properties of the scale and comparing them to the properties of the VAS^a^	*N* = 104; 5–16 years; Routine check-up/pain clinics; Healthy/recurrent headache	There was no significant difference in pain scores between the VAS and CAS for the same event. Higher mean scores were reported for severe tissue damage injuries such as broken bones than for minor bruises. 87% found the CAS very easy to use whereas 22% found the VAS easy to use	70
Miro (2004) [41]; Spain; Quantitative; Prospective; Acceptability, construct validity, reliability	1) determine the initial psychometric properties of the Catalan version of the FPS-R^c^ 2) compare patients’ opinion of the FPS-R with the CAS^d^	*N* = 371; Hospital/school; 7–15 years; Hospitalised/healthy	Correlations between the FPS-R and CAS ranged from r = 0.83–0.9. Relationship between pain and affective state r = 0.32. Test–retest ranged from r = 0.26–0.7. The proportion of children that preferred the FPS-R was significantly higher than the proportion that preferred the CAS (66–68%)	46
Morley (2014) [42]; Canada; Qualitative—cognitive interviews; Cross-sectional; Feasibility, acceptability	To cognitively test the Pediatric Advanced Care Quality of Life Scale (PAC-QoL) to establish whether the items and response options were understood	*N* = 34; Hospital; 8–18 years; Oncology;	Response scale was accurately interpreted in 88–93% of cases. When participants had trouble distinguishing between responses it involved options in the middle of the 4-point scale (sometimes and often)	65
O’Sullivan (2014) [43]; Canada; Qualitative; Cross-sectional; Feasibility	To evaluate and refine a new instrument for cancer symptom screening (SSPedi), including evaluating understanding of the response scale	*N* = 30; Hospital; 8–18 years; Oncology	Response options (5-point Likert) were understood by 90% of children	60
Ogden (2008) [44]; UK; Mixed methods; Cross-sectional; Acceptability, feasibility	To identify changes needed to adapt the IMPACT instrument for use in British children with inflammatory bowel disease and to see whether children preferred the Likert scale or the VAS^a^	*N* = 20; Outpatients; 8–16 years; Gastroenterology	Participants distinguished between the responses in the Likert scale and related their answers to the response options proficiently. Some children only guessed that ‘moderate’ meant ‘in the middle’ because of its position in the scale (5 point). 75% preferred the Likert scale to the VAS as it was easier and quicker to complete (p < 0.01)	55 quant 45 qual
Okanda (2010) [45]; Japan; Quantitative; Cross-sectional; Feasibility	To investigate whether 3–6-year-old children exhibit a ‘yes’ bias to various yes–no questions and whether their knowledge status affects the production of a yes bias	*N* = 135; Kindergarten/ nursery; 3–6 years; Healthy	3-year-olds had a strong tendency to exhibit a yes bias to both preference-object and knowledge object yes–no questions (even though they know the answer, p < 0.01). 4-year-olds could appropriately answer preference questions but showed a yes bias to knowledge questions (p < 0.1). 5- and 6-year-olds did not show a response bias to yes questions but showed a weak tendency to say yes to knowledge questions regarding familiar objects	55
Ortqvist (2012) [46]; USA; Qualitative; Cross-sectional; Feasibility	To examine how well the Knee Injury Osteoarthritis Outcome Score for Children (KOOS-Child) is understood	*N* = 34; Outpatient clinics; 10–16 years; Knee injury	Most children understood how to use a 5-point Likert response scale. The response option ‘moderate’ was persistently perceived as confusing. Most could interpret the meaning of the word by its location in the scale but could not define the word and suggested replacing it with the word ‘some’	70
Pagé (2012) [56]; Canada; Quantitative; Prospective; Acceptability, construct validity, feasibility	To evaluate the convergent and discriminant validity of the NRS^b^ for pain intensity and unpleasantness in children after surgery	*N* = 83/69; Hospital; 8–18 years; Orthopaedic/general surgery	The NRS correlated highly with the VRS^h^ and FPS-R^c^ (p < 0.001). Scores were significantly higher at 48–72 h post-surgery than at 2 weeks (p < 0.001). Children found the faces scale easiest to use (51%). The VRS was least liked (13%) and hardest to use	82
Rebok (2001) [92]; USA; Qualitative—cognitive interviews; Cross-sectional; Acceptability, feasibility	(1) to determine whether children can answer health survey items. (2) to test the feasibility of a pictorial questionnaire format using cartoon drawings of a child. (3) to examine several types and numbers of response formats to see which are preferred and most easily understood. (4) to test children’s understanding of specific concepts of health and wording of different response formats	*N* = 114; School/kindergarten; 5–11 years; Healthy	74% preferred circle responses to VAS^a^, with 68% preferring graduated circles. 74% preferred 4 rather than 3 circles. 100% preferred a horizontal presentation. Younger children gave a significantly higher number of extreme responses. Younger children effectively reduced a 5-point response format to 3 points by using only the middle and extremes. 67% preferred the 5-point response format (rather than 4 point)	70
Shields (2003) [47]; USA; Quantitative; Cross-sectional; Feasibility	To identify demographic and cognitive variables that would maximise the accuracy of predicting children’s abilities to use a VAS^a^	*N* = 40; Kindergarten; 5–7 years; Healthy	Only 42% of participants could use a VAS. Cognitive ability (IQ ≥ 100) combined with chronological age (≥ 5.6 years) was the best predictor of accurate use	80
Shields (2005) [48]; USA; Quantitative; Cross-sectional; Feasibility	To determine whether age, combined with estimated IQ, is an accurate predictor of a child’s successful use of a VAS^a^ in a non-clinical situation vs an acute, clinically emergent situation	*N* = 104; Hospital; 5–11 years; Healthy/lacerations	Estimated IQ and the ability to do a seriation task were the best predictors of 5–6-year-olds ability to accurately use the VAS (p < 0.001). Estimated IQ was not as important as chronological age and ability to perform a seriation task in those 7 years and over	83
Stanford (2006) [49]; Canada; Quantitative; Cross-sectional; Feasibility	To examine variations in 3- to 6-year-old children’s ability to accurately use a common self-report scale to rate pain in hypothetical vignettes (faces pain scale revised)	*N* = 112; Community; 3–6 years; Healthy	5- and 6-year-old children were significantly more accurate (40% errors) in their use of the FPS-R^c^ in response to the vignettes than 4-year-old children, who in turn were significantly more accurate than 3-year-old children (60% errors). Over half of 6-year-olds demonstrated difficulty using the FPS-R in response to the vignettes. Child age was the only significant predictor of children’s ability to use the scale in response to the vignettes (p < 0.001). The ability to use the scale improved with age	65
Staphorst (2017) [50]; Netherlands; Mixed methods; Cross-sectional; Acceptability, construct validity feasibility	To develop a generic, short and child-friendly instrument: the DISCO-RC questionnaire (DISCOmfort in Research with Children)	*N* = 46; Outpatients; 6–18 years; Unclear	Children preferred a 5-point Likert scale as a response option. The 5-point Likert scale coloured numeric VAS^a^ and simple VAS were strongly correlated (r = 0.76 – 0.99)	60 (quant) 65(qual)
Tesler (1991) [51]; USA; Quantitative; Cross sectional; Acceptability, construct validity, reliability, responsiveness	A program of studies designed to select and test a pain intensity scale for inclusion in a multidimensional pain assessment tool for children, focusing on determining each scale’s reliability, validity, ease of use and preference.5 scales were tested: a word graphic scale. VAS^a^, graded graphic rating scale, 0–10 magnitude estimation scale and CAS^d^	*N* = 1223; Hospital, outpatient, school; 8–18 years; Acute/healthy/chronic illness	Convergent validity for the 5 scales was supported (r = 0.66–0.84). The word graphic rating scale (Likert) was preferred by 47% of sick children. When used in a multidimensional pain assessment tool it showed test–retest reliability (r = 0.68–0.97) also showed sensitivity to change (p = 0.002)	65
Tomlinson (2019) [93]; Canada; Qualitative; Cross-sectional; Feasibility	To develop a new self-report symptom screening tool for children receiving cancer treatments who are 4–7 years of age (mini-SSPedi), based on SSPedi	*N* = 100; Hospital; 4–7 years; Oncology	Dichotomous response scale (yes/no) was understood by all participants. 80% understood the Wong-Baker faces, 70% understood FPS-R^c^ and 65% understood the pieces of hurt scales	60
van Laerhoven[59] (2004); Netherlands; Quantitative; Cross-sectional; Acceptability, feasibility	To examine which response options children prefer and which they find easiest to use (VAS^a^ vs Likert). To examine the relative reliability of the different response options	*N* = 122; Outpatients; 6–12 years; Not specified	Children preferred the Likert scale. They considered the Likert scale easiest to fill out. Results of the different response options correlated strongly with each other (r = 0.67– 0.90)	59
von Baeyer (2013) [52]; Canada; Quantitative; Cross sectional; Feasibility	To evaluate a binary question followed by simple response options for pain assessment in young children (FPS-R)	*N* = 184; Preschool/day care; 3–5 years; Healthy	3- and 4-year-olds performed significantly better using the simplified task than the FPS-R^c^ (p < 0.001). The simplified pain task made no difference to the 5-year olds who had almost identical mean scores using both methods. Response bias is common in children under 5	68
Vreeman (2014) [94]; Kenya; Qualitative—cognitive interviews; Cross-sectional; Acceptability, feasibility	To improve the understandability of paediatric antiretroviral adherence measurement items through cognitive interviewing with paediatric caregivers and HIV-infected adolescents	*N* = 10; HIV clinic; 13–18 years; HIV	Participants inconsistently quantified the differences between 4-point Likert response options. Visual analogue scales and the addition of a response option to give 5-points yielded more divergence and were considered hard to understand. It was suggested that VAS^a^ would require pictorial cues to orientate the participant to scale meaning	70
Watson (2006) [53]; USA; Quantitative; Cross-sectional; Feasibility	To evaluate the psychometric properties of the fruit and vegetable self-efficacy (FVSEQ) questionnaire	*N* = 1477; School; 9–10 years; General	Item response modelling showed that the 5-point response scale was not fully utilised	86
West (1994) [54]; USA; Quantitative; Cross-sectional; Feasibility, construct and convergent validity	To identify a clinically feasible and accurate method of measuring pain intensity in paediatric oncology patients in the ITU (FPS and Poker chip)	*N* = 30; Intensive care; 5–13 years; Oncology	Pain rating scales on the two tools were correlated (faces pain scale and Poker Chip, r = 0.67). 91.6% preferred the faces pain scale to the poker chip tool	50

**Table 3:** Summary of studies on recall period

Author (date); Country; Study Design; Measurement properties evaluated	Objective	Sample size (*N*); Setting; Age; Population	Main findings	QualSyst Score (%)
Chogle (2012) [61]; USA; Quantitative; Prospective; Acceptability, feasibility	To assess ability to accurately recall abdominal pain in children—comparison of daily reports vs one-month recall	*N* = 63; Outpatients; 8–17 years; Functional gastro-intestinal disorders	Most children reported a lower frequency of abdominal pain by recall than daily diaries (r = 0.4; CI 0.17–0.59%). Children 8–11 years had a higher correlation (r = 0.59) than those 12–18 (r = 0.26). Similar correlations were found to just the past 7 days (r = 0.47)	68
Heyer (2014) [62]; USA; Quantitative; Prospective; Feasibility, reliability	To compare the 90 day and 30-day recall of paediatric migraine disability assessment (PedMIDAS) elements and headache frequency against daily entries from an internet headache diary	*N* = 52; Outpatients; 10–18 years; Migraine	Reliability improved at 30-day recall compared to 90 days. 90-day diary: PedMIDAD r = 0.65; headaches r = 0.8330-day diary: PedMIDAD r = 086; headaches r = 0.88. Age and confidence in ability to answer were poor predictors of recall accuracy	86
Irwin (2009) [95]; USA; Qualitative; Cross-sectional; Feasibility	To conduct cognitive interviews with children and adolescents to gain feedback on items measuring physical functioning, emotional health, social health, fatigue, pain and asthma specific symptoms for PROMIS item bank	*N* = 100; Hospital; 4–7 years; Oncology	All children reported that the 7-day recall period meant the past 7 days and responded to items accordingly	60
Jacobson (2015) [67]; USA; Qualitative; Cognitive interviews; Cross-sectional; Feasibility	To develop and evaluate item candidates for new PROMIS Pediatric Pain Quality and Pain Behavior item banks, and Pain Intensity items	*N* = 34; Hospital; 8–18 years; Chronic pain	Participants from 8–18 years old understood that the recall period referred to the past week. There was a need to reiterate the 7-day time period to some younger children	70
Okupa (2013) [63]; USA; Quantitative; Prospective; Feasibility, reliability	To compare daily diaries vs retrospective questionnaires to assess asthma control	*N* = 88; Asthma Research and Education Network Centres; 6–17 years; Asthma	Asthma control days correlated better with daily diary information from the last 2 weeks of a 4-week recall (r = 0.46) than from the first 2 weeks	68
Ravens-Sieberer (2014) [66]; USA; Qualitative; Cognitive interviews; Acceptability and feasibility	To (1) conceptualize children’s subjective well-being and (2) produce item pools with excellent content validity for calibration and use in computerized adaptive testing	*N* = 37; Not stated; 8–17 years; Healthy and chronic conditions	Cognitive interviews supported children’s capacity to use a 7-day recall period for positive affect and a 4 week recall period for life satisfaction	65
Rebok (2001) [92]; USA; Qualitative—cognitive interviews; Cross-sectional; Acceptability, feasibility	1) to determine whether children can answer health survey items. 2) to test the feasibility of a pictorial questionnaire format using cartoon drawings of a child. 3) to examine several types and numbers of response formats to see which are preferred and most easily understood. 4) to test children’s understanding of specific concepts of health and wording of different response formats	*N* = 114; School/kindergarten; 5–11 years; Healthy	80% of participants could accurately use a 4 week recall period. Younger children did not understand the concept of a week and may not have used the 4-week time interval appropriately	70
Self (2015) [64]; USA; Quantitative; Prospective; Feasibility, reliability	To evaluate correspondence between retrospective questionnaire and prospective diary data for children and adolescents with IBS	*N* = 50; Outpatients; 8–18 years; Irritable bowel	For pain days ICC = 0.83 and days without bowel movement ICC = 0.74. Maximum pain score ICC = 0.8 and days with diarrhoea = -0.03. Although under conditions likely to facilitate agreement and with individual variation observed. Age was not significantly related to difference scores	70
Tomlinson (2019) [93]; Canada; Qualitative—cognitive interviews; Cross-sectional; Feasibility	To develop a new self-report symptom screening tool for children receiving cancer treatments who are 4–7 years of age (mini-SSPedi), based on SSPedi	*N* = 100; Hospital; 4–7 years; Oncology	Only 40% understood the time frame yesterday, so today was chosen for the measure	60
van den Brink(2001) [65]; Netherlands; Quantitative; Prospective; Feasibility, reliability	To investigate whether children and adolescents can recall prior headache complaints accurately and to study whether age, gender, headache severity, preferred coping strategies, depression, somatization, and trait anxiety are related to recall errors, causing recall bias	*N* = 100; School; 9–16 years; Headache	Compared to daily diary, retrospective questions led to overestimation of headache intensity and duration (r = 0.16). Lower age and increased headache severity were statistically related to recall errors	50
Vreeman (2014) [94]; Kenya; Qualitative; Cross-sectional; Acceptability, feasibility	To improve the understandability of paediatric antiretroviral adherence measurement items through cognitive interviewing with paediatric caregivers and HIV-infected adolescents	*N* = 10; HIV clinics; 13–18 years; HIV	Adolescents preferred either a 24-h recall period for ease of remembering or a 1 month recall as clinic appointments were monthly	70

**Table 4:** Summary of studies on administration mode

Author (date); Country**;** Study Design; Measurement properties evaluated	Objective	Sample size (*N*)**;** Setting; Age**;** Population	Main findings	QualSyst Score (%)
Bender (2007) [68]; USA; Quantitative; Prospective; Reliability	To test the effect of reporting mode on accuracy of inhaled cortico-steroid adherence reporting in children with asthma and their parents under conditions similar to those of an asthma clinical trial	*N* = 104; Outpatients; 8–18 years; Asthma	All methods led to over-reporting compared to electronic device on asthma pump. More than half of children over- reported adherence by > 25% Discrepancy was greatest in computer interview condition	77
Castarlenas (2015) [69] Spain; Quantitative; Cross-sectional; Acceptability, construct validity	To examine the agreement between verbally and electronically administered NRS-11^b^ (eNRS) for pain	*N* = 191; School; 12–18 years; Healthy	Bland Altman LOA fell outside the a priori limit for 95%. LOA at 80% fell inside the maximum limit established a priori. K-coefficients ranged from 0.786–0.912 indicating almost perfect agreement. 83% preferred the eNRS	77
Eaton (2010) [89]; USA; Quantitative; Cross-sectional; Construct validity, feasibility	To examine whether paper and pencil surveys and web surveys yield equivalent risk behaviour prevalence estimates when using the Youth Risk Behaviour Survey	*N* = 5227; School; Unclear; Healthy	Prevalence estimates from paper and pencil and web-based surveys were generally equivalent. Questionnaire mode was only significantly (p < 0.05) associated with 7 of 74 risk behaviours	82
Fouladi (2006) [70]; USA; Quantitative; Cross-sectional; Construct validity, feasibility	To examine systematic differences in the responses of 4th, 5th, and 6th graders to measures of stress, coping, and humour among three modes of assessment: paper-and-pencil questionnaires, computer-assisted self-interviewing (CASI), or a combination of paper-and-pencil and CASI. Scales used – feel bad scale, school agers coping strategies inventory, the multi-dimensional sense of humour scale	*N* = 1245; School; 9–12 years; General	CASI means and medians were higher (p < 0.002) and correlations between CASI measures tended to be lower than those obtained with paper and pencil and mixed modes. CASI variances were lower	65
Geerdink (2009) [71]; Netherlands; Quantitative; Cross-sectional; Acceptability, construct validity, feasibility	To develop a reliable and user-friendly digital child health assessment questionnaire (CHAQ) to complete systematically at the outpatient paediatric rheumatology clinic	*N* = 51; Outpatients; Unclear; Juvenile arthritis	Correlation between the digital and paper versions was high (r = 0.974). No statistically significantly differences in median outcome were found in visual analogue scale (VAS) pain (25.6 vs 25.9 mm) and VAS well-being (20.1 vs 19.5 mm). Although the mean time (5.06 min) to complete the digital CHAQ was significantly longer than the mean time (3.75 min) to complete the paper form, the majority of patients (75%) preferred the digital version. User-friendliness received maximum positive score	59
Jensen (2010) [72]; Denmark; Quantitative; Prospective; Acceptability	To examine the assessments and priorities by children and adolescents of health care in a paediatric outpatient clinic, to examine the influence of the time factor on assessments and priorities by children and adolescents of health care, and to determine their preferred method of evaluation	*N* = 346; Outpatients; 11–17 years; Range of diagnoses	50.1% of children and adolescents preferred to complete an electronic questionnaire to a paper one. They did not want to receive questionnaires by email	45
Jones (2010) [73]; New Zealand; Quantitative; Prospective; Acceptability, construct validity, reliability	To investigate the reliability and validity of a computerised anxiety assessment (smiley faces program revised (SFP-R)) and to explore children’s preferences for the method of anxiety assessment	*N* = 206; School; 5–13 years; Healthy	The online SFP-R demonstrated good reliability (⍺ = 0.75) and strong convergent validity with the modified children’s dental anxiety scale (r = 0.75). Test–retest reliability r = 0.67. Children preferred the computerised assessment to pen and paper methods	54
Knight (2007) [74]; USA; Quantitative; Cross-sectional; Acceptability	To determine adolescents’ preferences for method of substance abuse screening	*N* = 2133; Outpatients; 12–18 years; General medicine	Paper was the preferred method (mean rank (MR) = 2.92, 95%CI 2.87–2.96) vs. computer (MR = 2.38, 2.33–2.43), nurse (MR = 2.43, 2.39–2.47), and doctor (MR = 2.30, 2.25–2.35). Participants stated they were more likely to be honest with paper followed by computer, rather than responding to questions administered by a doctor or nurse. Those reporting on the computer were significantly more likely to report drug and alcohol use	67
Lloyd (2011) [75]; UK; Quantitative; Cross-sectional; Construct validity, feasibility, Reliability,	To examine the psychometric properties of an Internet version of a children and young persons’ quality of life measure (Kid’s Life and Times) originally designed as a paper questionnaire	*N* = 3440; School; 10–11 years; Healthy	Exploratory principal component analysis supported 5 components, in line with the paper version. Items loaded on to the expected components. Internal consistency was similar to that reported for the paper version (⍺ all > 0.76). Domain scores were similar to those reported in the literature for the paper version. Non-response was lower with the online version (1% vs 1.72–3.83%)	72
Magnus (2016) [90]; USA; Quantitative; Cross-sectional; Construct validity	To test the equivalence of scores obtained with the PROMIS paediatric depressive symptoms, fatigue and mobility measures across computer and telephone administration	*N* = 377; Home; 8–17 years; Healthy	There were high correlations between the two modes of administration (0.71–0.94), although fatigue scores were affected by mode of administration, but the differences in scores were sufficiently small that they would not affect overall interpretation of results	77
Mangunkusumo (2005) [76]; Netherlands; Quantitative; Cross-sectional; Acceptability, construct validity	To assess whether scores of an internet administered adolescent health questionnaire (KIVPA) are equivalent to those obtained via paper and pencil. To compare adolescents’ evaluation of administration modes	*N* = 565; School; 13–17 years; Healthy	Internet questionnaire generally resulted in equal scores to pen and paper mode. Adolescents in the internet one-item mode group more frequently reported satisfaction with appearance compared with the Internet multiple items mode (p ≤ .01). The internet group had more adolescents reporting that they had a sufficient number of friends compared to the paper mode (p ≤ .01)	77
Mangunkusumo (2006) [77]; Netherlands; Quantitative; Cross-sectional; Construct validity, feasibility	To compare the feasibility, presence of score differences and subjective evaluations by children between Internet and identical paper questionnaires (International study of asthma and allergies in childhood questionnaire)	*N* = 249; School; 10–12 years; Healthy	There were similar mean scores between administration modes. ICC 0.64–0.9. One third of items showed moderate agreement between modes (kappa 0.43–0.6). The remaining items had very good agreement (kappa 0.61–0.95). There were fewer missing data with the internet version	82
Mauz (2018) [78]; Germany; Cross-sectional; Acceptability, construct validity, feasibility	To determine whether prevalence rates or mean values of self-reported health indicators for children and adolescents age 11–17 years differ between self-administered paper-based questionnaires and self-administered web-based questionnaires (German Health Interview and Examination Survey for Children and Adolescents)	*N* = 1194; Home; 11–17 years; Healthy	Most questions showed mode equivalence except for alcohol consumption. Higher levels of consumption were reported online (p < 0.001). Male adolescents preferred the online mode. Those choosing the web-based response format were more likely to have higher household income and higher educational attainment (actual data not reported)	71
McCabe (2005) [79]; USA; Quantitative; Cross-sectional; Construct validity, feasibility	To examine the feasibility and mode effects of using a web form vs paper form survey to collect alcohol and tobacco data from 3rd and 4th grade students	*N* = 323; School; Not specified (3/4 grade); Healthy	There were minimal differences between survey modes. (future alcohol use and lifetime alcohol use showed significant difference, p < 0.05))	55
Moskowitz (2004) [80]; USA; Quantitative; Cross-sectional; Construct validity, feasibility	To assess the effect of telephone audio computer-assisted self-interviewing (A-CASI) and computer-assisted telephone interviewing (T-ACASI), on self-reports of smoking behaviour and smoking susceptibility among adolescents 12–17 years of age (adapted from Youth Attitudes and Practices Survey)	*N* = 2444; Home; 12–17 years; Healthy	Adjusted estimates of current smoking were higher in the self-administered T-ACASI (8.3% vs 4.5%). The commitment not to smoke among those who had never smoked was also higher in the T-ACASI (45% vs 34.9%). Parental presence was negatively associated with smoking. T-ACASI survey had more missing data than CATI	77
Nitikman (2017) [81]; Canada; Quantitative; Prospective; Construct validity, feasibility, reliability	To validate and test the reliability of using the Internet as a method of administering health-related quality of life questionnaires in a paediatric spine population (Scoliosis Research Society 30 (SRS-30) and Pediatric Outcomes Data Collection Instrument (PODCI))	*N* = 96; Outpatients; 11–18 years; Scoliosis	There was no significant difference in scores between methods of administration at the 2 time points (p = 0.206). Patients expressed a preference for the internet option (84%)	63
Raat (2007) [82]; Netherlands; Quantitative; Cross-sectional; Construct validity, feasibility, reliability	To evaluate the indicators of feasibility, reliability and validity of the Child Health Questionnaire-Child Form (CHQ-CF). To compare the results in those of those who complete the standard paper version compared to an internet version	*N* = 933; School; 13–17 years; Healthy	The internet version resulted in fewer missing answers. All scales clearly discriminated between adolescents with no, a few or many self-reported chronic conditions. The paper administration resulted in statistically significant, higher scores on 4 of 10 CHQ-CF scales compared with the internet administration (P < 0.05), but Cohen’s effect sizes d were ≤ 0.21. Mode of administration interacted significantly with age (P < 0.05) on four CHQ-CF scales, but Cohen’s effect sizes for these differences were also ≤ 0.21	96
Raat (2007) [83]; Netherlands; Quantitative; Cross-sectional; Construct validity, feasibility	To compare the results from written and internet questionnaires about respiratory symptoms to find out if both forms yielded the same responses (International Study of Asthma and Allergies in Childhood (ISAAC) questionnaire)	*N* = 933; School; 13–17 years; Healthy	The Internet version showed fewer missing answers not statistically significant). The respiratory items did not show statistically significant score differences between the Internet and written modes of administration. Both approaches yielded equal results	96
Robles (2015) [84]; Spain; Quantitative; Cross sectional; Construct validity, feasibility,	To develop web-based Spanish and Catalan versions of the EQ-5D-Y, and to compare scores and psychometric properties with the paper version	*N* = 715; School; 8–18 years; Healthy	Both formats of EQ-5D-Y showed low percentages of missing values (*n* = 2, and 4 to 9 for web and paper versions respectively), and a high ceiling effect by dimension (range from 79 to 96%). Percent agreement for EQ-5D-Y dimensions on the web and paper versions was acceptable (range 89% to 97%), and k ranged from 0.55 (0.48–0.61, usual activities dimension) to 0.75 (0.68–0.82, mobility dimension). Mean score difference on the VAS was 0.07, and the ICC for VAS scores on the two formats was 0.84 (0.82–0.86). Both formats showed acceptable ability to discriminate according to self-perceived health, reporting chronic conditions, and mental health status	83
Sun (2015) [91]; Canada; Quantitative; Longitudinal; Acceptability, construct validity, feasibility	To evaluate agreement between electronic (called Panda) and paper versions of the faces pain scale revised (FPS-R) and colour analogue scale (CAS)	*N* = 62; Hospital; 4–18 years; Surgical	Panda scores correlated strongly with original scores at T0 and T30 (r > 0.93 for FPS-R; r > 0.87 for CAS). Most participants expressed a preference for the iPod Panda version (76–81%)	67
Trapl (2013) [85]; USA; Quantitative; Cross sectional; Acceptability, feasibility	To examine the impact of 3 data collection modes (paper, PDA, audiPDA (APDA)) on the number of questions answered, data quality, and student preference	*N* = 275; School; Not specified (7^th^ grade); Healthy	APDA respondents completed significantly more questions compared to paper and PDA (p < 0.001). PDA and APDA had significantly fewer missing data than did paper (p < 0.001). No differences were found for student evaluation	63
Varni (2009) [86]; USA; Quantitative; Cross-sectional; Construct validity	To implement the multigroup confirmatory factor analysis (CFA) method for invariance testing across mode of administration for children’s self-reported health-related quality of life (in person, mail and telephone) using PedsQLTM 4.0 Generic Core Scales	*N* = 3741; Home or clinic; 5–18 years; Chronic illness	Strong factorial invariance across the mode of administration groups was demonstrated based on stability of the Comparative Fit Index (CFI) between the models, and several additional indices of practical fit including the Root Mean Squared Error of Approximation (RMSEA), the Non-Normed Fit Index (NNFI), and the Parsimony Normed Fit Index (PNFI). Children across the three modes of administration groups interpreted items on the PedsQLTM 4.0 Generic Core Scales in a similar manner	75
Wood (2011) [87]; France; Quantitative; Cross-sectional; Acceptability, construct validity, feasibility	To compare concordance and preference for electronic and paper versions of the faces pain scale revised, and to determine whether the electronic version can be used by children 4 years and over	*N* = 234; Hospital; 4–12 years; Inpatients	Overall weighted kappa = 0.846 and Spearman’s correlation between scores on the 2 versions was 0.91. The mean difference between scores was neither clinically nor statistically significant. 83.2% chose the same face on both versions. The PDA was preferred by 87.4% of participants	88
Young (2009) [88]; Canada; Quantitative; Prospective; Construct validity, feasibility	To test the impact of web administration on well-established measures of children’s physical function and quality of life using the ASK and PedsQL measures	*N* = 91 time 1 *N* = 69 time 2; Hospital; 8–14 years; Chronic illness	Both measures were highly reliable in web and paper format. Inter-method ICC = 0.98 for ASK and 0.64 for PedsQL compared to ICC of 0.99 and 0.94 respectively for paper formats. The web ASK seems to be valid compared to paper format. Consistency in administration mode may be more important when using the PedsQL	88

### Quality of included studies

Study quality ranged from 38 to 96%, with 10/81 scoring less than the 55% quality inclusion threshold recommended by the QualSyst [15]. The main reasons for poor scoring were small sample size, using parametric statistical tests without stating whether data was normally distributed, treating data from Likert scales as if it was interval, using Pearson’s correlation coefficient instead of intraclass correlation coefficient [97] and not stating randomisation methods. Qualitative papers rarely discussed reflexivity, the role of the researcher in the interview process or the connection to a theoretical framework. These low scoring studies were included in the review as it is often difficult to determine whether quality scoring elements were not reported rather than not taken into consideration.

### Response format

50 papers investigated ability to use specific response formats [16–60, 92–96] (see Table 2 for details). The majority reported on one or more of the following pictorial scales, (faces pain scale revised (FPS-R) or Wong-Baker faces) (*n* = 24), visual analogue scales (VAS) (*n* = 15), and Likert scales (numerical or word descriptor) (*n* = 14). The methodology for these studies was mainly quantitative, assessing acceptability, feasibility, validity and reliability. Nine qualitative studies used cognitive interviews to assess children’s understanding of response formats.

One study demonstrated that 3-year-olds exhibited a ‘yes’ bias to knowledge and preference-based questions even though they knew the answer should be ‘no’. By the age of 5–6 years this response bias did not exist in preference-based questions and was only weakly associated with knowledge questions regarding familiar objects [45].

### Pictorial scales (*n* = 24 studies)

Most pictorial scales for children are ‘faces’ scales. These are generally used for self-reporting pain and show a series of faces with graded intensity from ‘no pain’ to ‘worst pain possible’ [24]. Children are asked to point to the face that best shows how they are feeling. Most studies in this review have used either the Wong-Baker Faces scale (*n* = 5) or the FPS-R (*n*-19). The Wong-Baker scale has six cartoon-like, hand drawn faces ranging from smiling to crying with a score of 0–5 [98]. The FPS-R was adapted from the original FPS which had seven faces [99]. The FPS-R excludes smiles and tears and has six hand-drawn faces rather than seven so that it can be scored from 0 to 5 allowing scoring to be in line with other pain measures [32]. There is also a simplified version of the FPS (S-FPS), designed for children 3–5 years old, which first asks the child if they are in pain and if they respond ‘yes’ then they are shown a three-point faces scale [27].

From the age of seven, the use of six-point faces scales shows construct (convergent and discriminant) validity [16, 41, 49, 56, 96]. Convergent validity was found with numerical/verbal rating scales, VAS and the Poker Chip Tool in children 6–8 years old (*r* > 0.7 or *p* < 0.001) [22]). The Poker Chip (known as Pieces of Hurt) tool involves children being asked to pick the number of Poker Chips that represent their level of pain. One chip represents a small amount of pain and four the most amount.

Cognitive interview studies showed that children of 7 and over are generally able to understand and complete faces measures [96]. In younger children, the evidence on ability to use faces scales is mixed. Two studies report that six-point faces scales are valid (convergent validity *r* > 0.71 with word descriptor scale; discriminant validity *p* < 0.001 before and after a painful procedure) and reliable (test–retest reliability *r* = 0.9, *p* < 0.005) in children as young as three. These studies had relatively low quality scores and data on 3–7-year olds was analysed together [36, 57]. Other studies have shown that not all children under 7 years are able to understand six-point faces scales, and some have difficulty in using the middle of the scale [33, 49, 93, 96]. There is no evidence that ability to use faces scales differs between healthy children and those with underlying conditions.

Although faces scales tended to demonstrate convergent validity with other response formats such as VAS and the Poker Chip tool in children between 4 and 7 years, scores tend to be skewed low, suggesting children are scoring at the extremes and are unable to use the middle response option [31]. Studies of the S-FPS suggest that from 4 years, a three-point faces scale can be used reliably, although 4-year-olds tend to use the scale anchors thus rendering it dichotomous [26, 27].

Scales with smiling anchors lead to reporting of higher pain scores in 5–13-year-olds, compared to those with neutral face anchors, although scores between the two scales correlate [23–25]. Children aged 5–12 years expressed a preference for cartoon like faces in one study [24].

### Likert scales (*n* = 14 studies)

These studies were carried out with children 8 years and over, except one which had a lower age limit of 6 years [59]. Most showed that children from 8 years old can understand and use a 4 or 5-point Likert scale [20, 34, 42, 43, 46, 95], with scores correlating strongly with a VAS [59]. Cognitive interview studies (5–18 years) demonstrated that if children struggled with Likert scales, it was usually with the middle points of a scale [34, 42, 92] with the term ‘moderate’ being perceived as confusing [44, 46]. One study found that children 13–18 years old could not use a 4-point Likert scale as they were unable to quantify the differences between response options. Addition of a fifth point created more divergence and was harder to understand [94]. Four studies in children 8–18 years used item response theory to examine scale performance [17, 38, 53, 60]. Three found that using a five-point scale led to disordered thresholds and performance was enhanced by using a three-point scale [17, 38, 60]. One study in 9–10-year-olds showed that a five-point scale was not fully utilised [53]. Negatively formulated questions were shown to have no effect on reliability in one study [20]. As with faces scales, there is no evidence that ability to use a Likert scale differs between healthy and unwell children.

### Visual analogue scales (*n* = 15 studies)

A visual analogue scale is usually a 100 mm long horizontal line with verbal descriptors at each end expressing extremes of feeling. Respondents mark a point on the line that best corresponds to the severity of their symptom or feeling [100].

At all ages the VAS seems to be less valid and reliable to use than faces or Likert scales, with slight pain on a verbal rating scale corresponding to a wide interval of 7–65 on a VAS scale [18, 57]. In children aged 5–7 years, cognitive ability, chronological age and the ability to conduct a seriation task (arranging circles in order of size) seems to be the best predictor of ability to use a VAS [47, 48]. Cognitive ability was less important after the age of seven [48]. This finding is supported by a study in children 9–12 years with learning impairment who only used the scale anchors, whereas children without learning impairment of the same age were able to use the whole VAS [21]. One study suggests that for those over 8 years old, the addition of pictorial anchors allowed children to make greater use of the full scale [29].

### Other scales (*n* = 6)

The Pain Block Scale is a pictorial ordered block scale with a score between 0 and 10. This demonstrates agreement with the FPS-R and has discriminant validity in children from the age of 4–7 years who can count to five [35].

Two studies in children 3–14 years showed that the Poker Chip tool has convergent validity with faces scales (*r* = 0.67; *p* < 0.001) [30, 54] and one in children 4–7 years old showed convergent validity with VAS and VRS (*r* = 0.7) [31]. One study showed that 65% of 4–7-year olds understood the scale [93].

The coloured analogue scale (CAS) resembles a ruler, with one side showing a wedge-shaped figure filled with colour that progresses from white to red as the figure widens. The other side shows corresponding numerical ratings from 1 to 10 cm. One study demonstrated discriminant and construct validity with the VAS, and children from 5 to 16 years found the CAS easier to use than the VAS [55].

### Preference of scale (*n* = 13)

13 studies asked children 3–18 years their preference of scale [18, 22, 30, 36, 41, 44, 50, 51, 54–56, 59]. In all studies using a faces scale this was preferred to VAS and Likert scales [22, 30, 36, 41, 54, 56, 57]. In all but one study, Likert scales were preferred to VAS [36, 50, 51, 59]. Four studies examined preference for the CAS, and in three it was preferred to FPS-R, VAS and Likert scales [22, 51, 55]. The FPS-R was preferred to the CAS in one study [41].

### Recall period (*n* = 11)

11 studies reported on recall period [61–67, 92–95] (see Table 3 for details). Of these, 5/9 compared daily diary reports to retrospective questionnaires. Four of these were conducted in children 8 years and over and one in children from 6 years old. They showed that shorter recall periods lead to better correlation with daily diaries, with 7–14 days being optimal [61–65]. The other six studies were cognitive interview studies. These suggest that children under 8 years old cannot understand the concept of a week [92] and some could not understand the term ‘yesterday’ [93]. Those over 8 years could use both 7 day and 4-week recall periods [66, 67, 92, 95]. One study asked children 13–18 years old their recall preference and they suggested that 24 h was preferable but that one month would be easy to remember as they had monthly clinic appointments [94].

### Administration mode (*n* = 24)

24 studies reported on administration mode with children aged 4–18 years [68, 70–91, 96] (see Table 4). The majority compared paper and pencil PROMs with an identical computerised version. Most studies showed moderate to strong correlation between paper and computerised versions [71, 75, 76, 81, 83, 84, 87–89, 91]. All studies that asked preference for mode showed preference for computer-based measures [71–73, 78, 81, 87, 91]. Sensitive subjects such as stress, coping, alcohol and tobacco use were more likely to be reported using web-based measures in children 8–18 years [70, 74, 78, 79]. One study showed that those under 8 years needed help completing a computerised measure [96]. There was fewer missing data with computerised measures. It was not always clear whether this was due to the inability to move on until a question was completed [75, 82, 85]. Strong factorial invariance was found across telephone, face to face and mail [86], and computer and telephone methods were also shown to be strongly correlated [90].

## Discussion

This review provides evidence that CYP over 5 years old can meaningfully report on aspects of their own health, providing consideration is given to age, response format and recall period. CYP as young as 4 years old expressed a preference for completing measures regarding their health via a computerised method.

To self-report health-outcomes, children must have at least a rudimentary self-concept and ability to express this, understand the basic notions of health and illness, be able to pay attention, discriminate between the response options, recall health experiences and write a response [92]. Until 4–5 years old, children’s language and thought processes are limited, so their ability to go through these process is also limited [101]. Children as young as 3 years of age were included in some of the studies in this review but results were presented alongside those of children ranging from 6 to 17 years old. The results of this review suggest that most children over five are able to reliably self-report on their health to some degree, with children younger than this exhibiting a ‘yes’ bias in response to questions [45].

### Response format

Up until 6–7 years old, children view themselves in predominantly physical terms and their response to questionnaires is mainly dichotomous [102]. This is demonstrated in studies of 3–7-year-olds using a 3-point faces scale where only the anchors were used [26, 27]. Evidence on the ability of CYP over 7 years old to use 5- or 6-point response formats is mixed. This may be a reflection of variability in children’s development, with chronological age having less of an influence than cognitive ability [5]. Difficulty with the middle of scales was found in cognitive interview studies in those 5–18 years using Likert scales [42, 44, 92, 94]. In contrast, evidence from other cognitive interview and validity and reliability studies showed that those over 8 years old can understand 5-point Likert scales [20, 34, 42, 43, 46, 95] and that children over the age of 7 years can validly and reliably use scales with six faces [16, 33, 49, 93, 96]. However, item response theory studies show that the use of 5-point Likert scales led to disordered thresholds and 3-point scales functioned better in those 8–18 years old [17, 38, 60]. As data for all ages was usually presented together, it is not possible to ascertain whether older children can reliably use a 5-point response format. The VAS was less reliable and valid than Likert or faces across the age span [18, 57] and functions better with pictorial anchors [29]. There was an overwhelming preference at all ages for faces scales, with the VAS being the least preferred, suggesting that children are motivated by visually appealing response formats. It is recommended that when developing PROMS for CYP consideration is given to making them visually appealing to improve acceptability. It is also recommended that a dichotomous response format is used for those aged 5–7 years and a 3-point response format should be considered for those seven and over. Validity of response formats should not be evaluated solely in terms of convergent and discriminant validity of the measure, as this will often be high. Cognitive interview studies should also be undertaken, to give greater insight into how response format is understood. This review found no evidence that children who had underlying health conditions, were able to more reliably use any of the response formats described than their healthy peers.

### Recall period

Evidence on recall period is limited, with only 11 studies reporting on this. These suggest that recall period should be kept to 24–48 h for those under 8 [92, 93]. Those over 8 years old are able to respond reliably to events that occur over the past 7–14 days [66, 67, 92, 95]. It is recommended that when developing PROMs for CYP the recall period is kept to no more than 48 h for those under 8 years. From 8 years old CYP seem to be able to recall the past 14 days, but due to data being presented for wide age ranges is unclear from what age CYP may be able to recall further than this.

### Administration mode

Online and paper-and-pencil response formats demonstrated moderate to strong correlation [71, 75, 76, 81, 83, 84, 87–89, 91], similar to findings in adults [103] and there was an overwhelming preference for a computerised format [71–73, 78, 81, 87, 91]. Sensitive questions are more likely to be answered honestly in a computerised measure, probably as this method of data collection is perceived as more anonymous [70, 74, 78, 79]. There was fewer missing data on computerised versions of measures, possibly because children were not allowed to move to the next question if a response was left unanswered [75, 82, 85]. Those under 8 years old may need help from an adult to complete computerised outcome measures [96]. It is recommended that PROMS developed for CYP of all ages include a computerised version to enhance acceptability.

## Strengths and limitations

This systematic review provides evidence of children’s ability to self-report on their health outcomes in terms of recall period, response format and administration mode of measures but has some limitations. The inclusion criteria only incorporated articles published in the English language and searches were carried out in health-related databases; further evidence may be found in educational research. There were relatively few studies on recall period (*n* = 11) and the effects of cognitive ability rather than chronological age (*n* = 2) which highlight areas for future research. This review identified 13,215 articles for screening, another eight were included as a result of hand-searching and communication with experts. The assessment of recall period, response format and administration mode was a small part of these studies and as such, was not included in the paper keywords. The quality of included studies was poor in some instances which could have affected the reported results. These were included as it is often not possible to assess which aspects were addressed but not reported in the published paper. This is particularly relevant for older studies that were published before current reporting guidance was developed. Sample size was sometimes small, but it is well known that recruiting to paediatric research, particularly when this includes children with an underlying health condition, can be challenging [104]. A large number of studies were researching pain focused measures, rather than having a multi-dimensional focus.

Most included studies did not stratify their results by age, presenting data for wide age ranges. This makes it impossible to distinguish variation in ability by age group. As cognitive ability usually improves with age, it is recommended that when developing PROMs, psychometric testing is stratified by age and/or cognitive ability. PROM developers should also consider having different versions for different age groups or developmental ability to account for this. Future research could also take further steps to appraise the reliability of CYP self-report by using multi-indicator approaches, such as lack of response variability, excessive response variation and extreme, inconsistent or improbable response patterns, to assess invalid responses at the individual level [105].

Implications for developing PROMS for CYP.

From this systematic review we make eight recommendations for developing PROMS for CYP. These are:

1. Proxy measures should be used for those under 5 years old.
2. Measures should be visually appealing, to improve acceptability.
3. PROM studies should be analysed and reported in developmentally appropriate age bands.
4. Developers should consider different versions of a measure for different age groups.
5. Development should include both cognitive interview studies, and psychometric testing to enhance understanding of how children formulate answers.
6. 5–7 years olds should be given a dichotomous response format; those 7 years and over should be given a three-point response format.
7. Recall period should be kept short, no more than 48 h for those 5–7 years.
8. PROMS should have a computerised version.

We propose that these recommendations are used alongside the COSMIN and Rothrock [14, 106] guidance on PROM development and validation.

## Conclusion

Development of PROMS for CYP is complex and challenging due to diversity in developmental stage and cognitive ability. Children < 5 years old are unable to reliably report on their own health outcomes. Children < 8 years old cannot accurately recall beyond the past 48 h and can only reliably use a dichotomous response format. Children find visually appealing measures, in a computerised format more acceptable to use. Future work should focus on the impact of cognitive ability on self-report in CYP, reporting results of validation studies in smaller age ranges and establishing whether CYP with underlying health conditions are more able to report on their own health outcomes than their healthy peers. The results of this review have both clinical and research implications. They can be used to inform appropriate choice of PROM in the clinical setting. Our eight recommendations for developing PROMS for CYP can be used to further research in PROM development for CYP.

## Supplementary Information

Below is the link to the electronic supplementary material.

Supplementary file1 (DOCX 21 KB)

Supplementary file2 (DOCX 46 KB)

Supplementary file3 (DOCX 24 KB)

## Data Availability

The data that supports the findings of this review are available in the supplementary material.
