# Supplementary material for: Enhancing validity, reliability and participation in self-reported health outcome measurement for children and young people: a systematic review of recall period, response scale format, and administration modality
Source: Qual Life Res. 2021 Mar 18;30(7):1803–32. doi: 10.1007/s11136-021-02814-4 (PMC8233251; doi:10.1007/s11136-021-02814-4)
Supplement: Supplementary file 1 — Supplementary file1 (DOCX 21 KB) [file 11136_2021_2814_MOESM1_ESM.docx]

Supplementary Appendix 1. Full search strategy

##### Children

1 exp child/

2 exp p?ediatrics/

3 (child* or adolescen* or p?ediatric* or youth* or juvenile or teen* or young people or schoolchild* or school age* or kid*).ti,ab.

4 1 or 2 or 3

##### Response Scale format

5 (response scale or likert scale or visual analog* scale or VAS or numerical rating scale or verbal rating scale or faces scale or dichotomous scale or yes no response or response option*).ti,ab.

##### Recall period

6 (recall period or recall interval or patient recall or recall bias).ti,ab.

##### Method of administration

7 (outcome measure adj2 (paper or (paper and pen) or tablet or tablet computer or app or application or telephone or face to face or internet)).ti,ab.

8 (measure adj2 (paper or (paper and pen) or tablet or tablet computer or app or application or telephone or face to face or internet)).ti,ab.

9 (scale adj2 (paper or (paper and pen) or tablet or tablet computer or app or application or telephone or face to face or internet)).ti,ab.

10 (questionnaire adj2 (paper or (paper and pen) or tablet or tablet computer or app or application or telephone or face to face or internet)).ti,ab.

11 (survey adj2 (paper or (paper and pen) or tablet or tablet computer or app or application or telephone or face to face or internet)).ti,ab.

12 7 or 8 or 9 or 10 or 11

##### Combine the above

13 14 or 15 or 21

14 4 and 13 and 22

##### Exclusion criteria(21)

24 (addresses or biography or comment or directory or editorial or interview or festschrift or lectures or legal cases or legislation or letter or news or newspaper article or patient education handout or popular works or congresses or consensus development conference or practice guideline).pt

25 23 not 24

26 (limit to 1980-current; humans; English language; all child 0-18 years).
