# Supplementary material for: Enhancing validity, reliability and participation in self-reported health outcome measurement for children and young people: a systematic review of recall period, response scale format, and administration modality
Source: Qual Life Res. 2021 Mar 18;30(7):1803–32. doi: 10.1007/s11136-021-02814-4 (PMC8233251; doi:10.1007/s11136-021-02814-4)
Supplement: Supplementary file 2 — Supplementary file2 (DOCX 46 KB) [file 11136_2021_2814_MOESM2_ESM.docx]

Supplementary data 2. QualSyst Scores for Quantitative Studies

| Author | Objective | Design | Subject selection | Subject characteristics | Random allocation | Investigator blinding | Subject blinding | Outcome measures | Sample size | Analysis | Estimate of variance | Confounding | | Reporting of results | Conclusion | Score  (%) |
| --- | --- | --- | --- | --- | --- | --- | --- | --- | --- | --- | --- | --- | --- | --- | --- | --- |
| Baxter (2011)[16] | 1 | 1 | 1 | 1 | NA | NA | NA | 2 | 2 | 1 | 2 | | 0 | 2 | 2 | 68 |
| Bender (2007) [68] | 2 | 2 | 1 | 1 | 1 | 2 | NA | 2 | 1 | 2 | 2 | | 0 | 2 | 2 | 77 |
| Benson (2016)[17] | 0 | 2 | 1 | 1 | NA | NA | NA | 1 | 1 | 1 | 2 | | NA | 2 | 1 | 60 |
| Berntson (2001)[18] | 2 | 2 | 2 | 0 | NA | NA | NA | 2 | 0 | 2 | 2 | | 0 | 1 | 2 | 68 |
| Borgers (2003)[19] | 2 | 1 | 2 | 1 | NA | NA | NA | 1 | 2 | 1 | 2 | | NA | 2 | 2 | 75 |
| Borgers (2004)[20] | 2 | 1 | 2 | 1 | NA | NA | NA | 1 | 2 | 1 | 2 | | NA | 2 | 2 | 80 |
| Campbell (2011)[21] | 1 | 2 | 0 | 0 | NA | NA | NA | 2 | 0 | 2 | 2 | | 0 | 2 | 2 | 59 |
| Castarlenas (2013)[22] | 2 | 2 | 1 | 1 | NA | NA | NA | 1 | 2 | 2 | 0 | | NA | 2 | 2 | 75 |
| Castarlenas (2015)[69] | 2 | 2 | 1 | 1 | NA | NA | NA | 2 | 2 | 2 | 2 | | 1 | 2 | 0 | 77 |
| Chambers (1998)[23] | 2 | 1 | 1 | 0 | 1 | NA | NA | 2 | 1 | 1 | 2 | | 0 | 2 | 2 | 63 |
| Chambers (1999)[24] | 2 | 1 | 1 | 1 | 1 | NA | NA | 1 | 1 | 2 | 2 | | 2 | 2 | 2 | 75 |
| Chambers (2005)[25] | 2 | 2 | 1 | 1 | 1 | NA | NA | 2 | 2 | 2 | 2 | | 1 | 2 | 2 | 83 |
| Chogle (2012)[61] | 2 | 2 | 2 | 1 | NA | NA | NA | 2 | 1 | 0 | 2 | | 0 | 2 | 1 | 68 |
| Decruynaere (2009)[26] | 2 | 2 | 1 | 1 | NA | NA | NA | 1 | 1 | 2 | NA | | 0 | 2 | 2 | 70 |
| Eaton (2010)[89] | 2 | 2 | 1 | 0 | NA | NA | NA | 2 | 2 | 2 | 2 | | 2 | 2 | 1 | 82 |
| Emmott (2017)[27] | 2 | 2 | 2 | 2 | NA | 0 | NA | 2 | 2 | 1 | 2 | | 2 | 2 | 2 | 88 |
| Fanciullo (2007) [28] | 2 | 2 | 1 | 1 | NA | NA | NA | 2 | 1 | 1 | 2 | | NA | 1 | 2 | 75 |
| Fouladi (2006)[70] | 2 | 0 | 1 | 1 | 0 | NA | NA | 1 | 2 | 1 | 2 | | NA | 2 | 1 | 65 |
| Fritz (1994)[29] | 2 | 2 | 1 | 1 | NA | NA | NA | 2 | 1 | 1 | 2 | | 1 | 2 | 2 | 77 |
| Geerdink (2009)[71] | 1 | 1 | 1 | 1 | 1 | NA | NA | 2 | 1 | 1 | 2 | | NA | 1 | 1 | 59 |
| Gharaibeh (2002)[30] | 2 | 2 | 2 | 1 | NA | NA | NA | 1 | 1 | 1 | 0 | | NA | 1 | 1 | 60 |
| Goodenough (1997)[31] | 1 | 1 | 1 | 1 | 0 | NA | NA | 1 | 1 | 1 | 0 | | NA | 2 | 1 | 45 |
| Gulur (2009)[58] | 2 | 1 | 1 | 1 | NA | NA | NA | 2 | 1 | 1 | 2 | | NA | 1 | 0 | 60 |
| Heyer (2014)[62] | 1 | 2 | 2 | 2 | NA | NA | NA | 2 | 1 | 2 | 2 | | 1 | 2 | 2 | 86 |
| Hicks (2001)[32] | 1 | 1 | 0 | 0 | NA | NA | NA | 1 | 1 | 2 | 2 | | NA | 2 | 2 | 60 |
| Hunter (2000)[33] | 1 | 1 | 1 | 0 | NA | NA | NA | 2 | 0 | 1 | 1 | | 0 | 2 | 2 | 50 |
| Jensen (2010)[72] | 1 | 1 | 1 | 1 | 0 | NA | NA | 1 | 1 | 1 | 1 | | NA | 1 | 1 | 45 |
| Jones (2010)[73] | 1 | 1 | 1 | 1 | 0 | NA | NA | 1 | 1 | 1 | 2 | | 2 | 1 | 1 | 54 |
| Jung (2018)[35] | 2 | 2 | 1 | 1 | NA | NA | 2 | 1 | 1 | 1 | 2 | | NA | 1 | 1 | 68 |
| Keck (1996)[36] | 2 | 1 | 1 | 0 | NA | NA | NA | 1 | 1 | 1 | 0 | | NA | 1 | 1 | 50 |
| Klassen (2015)[60] | 2 | 2 | 1 | 2 | NA | NA | NA | 1 | 2 | 2 | 2 | | NA | 2 | 2 | 90 |
| Knight (2007)[74] | 1 | 1 | 1 | 2 | 0 | NA | NA | 1 | 1 | 2 | 2 | | 2 | 2 | 1 | 67 |
| Lawford (2001)[37] | 1 | 2 | 1 | 0 | NA | NA | NA | 1 | 1 | 1 | 2 | | NA | 2 | 2 | 65 |
| Leske (2015)[38] | 1 | 2 | 1 | 2 | NA | NA | NA | 1 | 2 | 2 | 2 | | NA | 1 | 2 | 80 |
| Lloyd (2011)[75] | 2 | 1 | 2 | 1 | NA | NA | NA | 2 | 2 | 1 | 2 | | 1 | 1 | 1 | 72 |
| Locker (2007)[39] | 2 | 2 | 1 | 1 | NA | NA | NA | 2 | 1 | 2 | 2 | | NA | 2 | 2 | 85 |
| Luffy (2003)[57] | 2 | 1 | 0 | 0 | NA | NA | NA | 2 | 1 | 1 | 0 | | NA | 2 | 1 | 50 |
| Magnus (2016)[90] | 2 | 1 | 1 | 2 | 1 | NA | NA | 2 | 2 | 1 | 2 | | NA | 2 | 1 | 77 |
| Maïano (2009)[40] | 2 | 0 | 2 | 0 | NA | NA | NA | 2 | 2 | 2 | 2 | | NA | 2 | 2 | 80 |
| Mangunkusumo (2005)[76] | 2 | 2 | 1 | 1 | 1 | NA | NA | 1 | 2 | 1 | 2 | | NA | 2 | 2 | 77 |
| Mangunkusumo (2006)[77] | 2 | 2 | 1 | 1 | 1 | NA | NA | 1 | 2 | 2 | 2 | | NA | 2 | 2 | 82 |
| Mauz (2018)[78] | 2 | 2 | 1 | 1 | 1 | NA | NA | 1 | 1 | 2 | 0 | | 2 | 2 | 2 | 71 |
| McCabe (2005)[79] | 1 | 1 | 1 | 1 | 1 | NA | NA | 1 | 1 | 1 | 0 | | 2 | 1 | 1 | 55 |
| McGrath (1996)[55] | 2 | 1 | 1 | 0 | NA | NA | NA | 2 | 1 | 1 | 2 | | NA | 2 | 2 | 70 |
| Miro (2004)[41] | 2 | 1 | 1 | 0 | 0 | NA | NA | 1 | 1 | 1 | 2 | | 0 | 1 | 1 | 46 |
| Moskowitz (2004)[80] | 2 | 1 | 1 | 2 | 1 | NA | NA | 2 | 1 | 0 | 2 | | 2 | 2 | 1 | 77 |
| Nitikman (2017)[81] | 2 | 1 | 1 | 1 | 2 | NA | NA | 1 | 0 | 1 | 2 | | 1 | 2 | 1 | 63 |
| Ogden (2008)[44] | 1 | 1 | 1 | 1 | NA | NA | NA | 2 | 0 | 1 | 2 | | NA | 1 | 1 | 55 |
| Okanda (2010)[45] | 1 | 1 | 1 | 0 | NA | NA | NA | 1 | 1 | 2 | 2 | | NA | 2 | 0 | **55** |
| Okupa (2013)[63] | 1 | 2 | 1 | 2 | 1 | 1 | 1 | 1 | 1 | 1 | 2 | | 1 | 2 | 2 | 68 |
| Pagé (2012)[56] | 2 | 2 | 0 | 2 | 2 | NA | NA | 2 | 1 | 1 | 2 | | NA | 2 | 2 | 82 |
| Raatt (2007)[82] | 2 | 2 | 1 | 2 | 2 | NA | NA | 2 | 2 | 2 | 2 | | 2 | 2 | 2 | 96 |
| Raat (2007)[83] | 2 | 2 | 1 | 2 | 2 | NA | NA | 2 | 2 | 2 | 2 | | 2 | 2 | 2 | 96 |
| Robles (2015)[84] | 2 | 2 | 1 | 2 | 1 | NA | NA | 2 | 2 | 2 | 2 | | 0 | 2 | 2 | 83 |
| Self (2015)[64] | 2 | 2 | 1 | 0 | NA | NA | NA | 0 | 1 | 2 | 2 | | NA | 2 | 2 | 70 |
| Shields (2003)[47] | 2 | 2 | 1 | 1 | NA | NA | NA | 1 | 1 | 2 | 2 | | NA | 2 | 2 | 80 |
| Shields (2005)[48] | 2 | 2 | 1 | 2 | 1 | NA | NA | 2 | 0 | 2 | 2 | | 2 | 2 | 2 | 83 |
| Stanford (2006)[49] | 1 | 1 | 0 | 1 | NA | NA | NA | 2 | 1 | 1 | 2 | | NA | 2 | 2 | 65 |
| Staphorst (2017) [50] |  |  |  |  |  |  |  |  |  |  |  | |  |  |  |  |
| Sun (2015)[91] | 2 | 2 | 1 | 1 | 1 | NA | NA | 1 | 1 | 1 | 1 | | 1 | 2 | 2 | 67 |
| Tesler (1991)[51] | 1 | 1 | 1 | 1 | NA | NA | NA | 1 | 2 | 1 | 2 | | NA | 1 | 2 | 65 |
| Trapl (2013)[85] | 1 | 1 | 1 | 1 | 1 | NA | NA | 1 | 1 | 1 | 2 | | 2 | 2 | 1 | 63 |
| van der Brink(2001)[65] | 2 | 1 | 0 | 0 | NA | NA | NA | 0 | 2 | 1 | 1 | | NA | 1 | 2 | 50 |
| van Laerhoven (2004)[59] | 2 | 1 | 1 | 1 | 0 | NA | NA | 2 | 1 | 2 | 0 | | NA | 2 | 1 | 59 |
| Varni (2009)[86] | 2 | 1 | 1 | 1 | 0 | NA | NA | 2 | 2 | 2 | NA | | 0 | 2 | 2 | 75 |
| von Baeyer (2013)[52] | 2 | 1 | 1 | 1 | 1 | NA | NA | 2 | 1 | 2 | 2 | | NA | 1 | 1 | 68 |
| Watson (2006)[53] | 2 | 2 | 1 | 1 | NA | NA | NA | 1 | 2 | 2 | 2 | | 2 | 2 | 2 | 86 |
| West (1994)[54] | 2 | 1 | 1 | 1 | NA | NA | NA | 1 | 1 | 1 | 0 | | NA | 1 | 1 | 50 |
| Wood (2011)[87] | 2 | 2 | 1 | 2 | 2 | NA | NA | 1 | 1 | 2 | 2 | | 2 | 2 | 2 | 88 |
| Young (2009)[88] | 2 | 2 | 1 | 2 | 2 | NA | NA | 2 | 1 | 2 | 2 | | 2 | 2 | 1 | 88 |
