# Supplementary material for: Enhancing validity, reliability and participation in self-reported health outcome measurement for children and young people: a systematic review of recall period, response scale format, and administration modality
Source: Qual Life Res. 2021 Mar 18;30(7):1803–32. doi: 10.1007/s11136-021-02814-4 (PMC8233251; doi:10.1007/s11136-021-02814-4)
Supplement: Supplementary file 3 — Supplementary file3 (DOCX 24 KB) [file 11136_2021_2814_MOESM3_ESM.docx]

Supplement 3 – QualSyst scores for qualitative studies

| Author | Objective | Design | Context | Connection to literature | Sampling | Data collection | Analysis | Credibility | Conclusions | Reflexivity | Score (%) |
| --- | --- | --- | --- | --- | --- | --- | --- | --- | --- | --- | --- |
| Gupta (2016) [96] | 1 | 2 | 1 | 1 | 1 | 2 | 1 | 0 | 2 | 0 | 55 |
| Irwin (2009) [95] | 2 | 2 | 2 | 0 | 2 | 2 | 1 | 0 | 2 | 0 | 65 |
| Jacobson (2015) [67] | 2 | 2 | 2 | 1 | 1 | 1 | 2 | 0 | 2 | 1 | 70 |
| Joffer (2016) [34] | 1 | 1 | 1 | 1 | 1 | 2 | 2 | 2 | 1 | 0 | 60 |
| Klassen (2015) [60] | 2 | 1 | 2 | 0 | 1 | 2 | 2 | 0 | 1 | 0 | 55 |
| Morley (2014) [42] | 2 | 2 | 2 | 0 | 1 | 2 | 1 | 2 | 1 | 0 | 65 |
| Ogden (2008) [44] | 1 | 1 | 2 | 1 | 1 | 1 | 1 | 0 | 1 | 0 | 45 |
| O'Sullivan (2014) [43] | 2 | 1 | 2 | 0 | 1 | 2 | 2 | 0 | 2 | 0 | 60 |
| Ortqvist (2012) [46] | 2 | 2 | 2 | 0 | 1 | 2 | 2 | 2 | 1 | 0 | 70 |
| Ravens-Sieberer (2014) [66] | 2 | 2 | 1 | 1 | 0 | 1 | 1 | 2 | 2 | 1 | 65 |
| Rebok (2001) [92] | 2 | 2 | 2 | 1 | 1 | 2 | 2 | 0 | 2 | 0 | 70 |
| Staphorst (2017) [50] | 1 | 2 | 2 | 1 | 0 | 1 | 2 | 2 | 2 | 0 | 65 |
| Tomlinson (2019) [93] | 1 | 2 | 2 | 1 | 1 | 1 | 2 | 1 | 1 | 0 | 60 |
| Vreeman (2014) [94] | 2 | 1 | 2 | 1 | 1 | 2 | 1 | 2 | 2 | 0 | 70 |
